# Structural Evidence for the Tetrameric Assembly of Chemokine CCL11 and the Glycosaminoglycan Arixtra™

**DOI:** 10.3390/biom3040905

**Published:** 2013-11-06

**Authors:** Andrew B. Dykstra, Matt D. Sweeney, Julie A. Leary

**Affiliations:** 1 Department of Obstetrics, Gynecology, and Reproductive Sciences, University of California, 521 Parnassus Avenue, San Francisco, CA 94143, USA; E-Mail: dykstraa@obgyn.ucsf.edu; 2 Perspectives, Inc., 2231 Garden Highway, Sacramento, CA 95833, USA; E-Mail: mattsweeneyphd@gmail.com; 3 Department of Molecular and Cellular Biology, University of California, 1 Shields Avenue, Davis, CA 95616, USA

**Keywords:** CCL11, ion mobility mass spectrometry, chemokine, glycosaminoglycan

## Abstract

Understanding chemokine interactions with glycosaminoglycans (GAG) is critical as these interactions have been linked to a number of inflammatory medical conditions, such as arthritis and asthma. To better characterize *in vivo* protein function, comprehensive knowledge of multimeric species, formed by chemokines under native conditions, is necessary. Herein is the first report of a tetrameric assembly of the human chemokine CCL11, which was shown bound to the GAG Arixtra™. Isothermal titration calorimetry data indicated that CCL11 interacts with Arixtra, and ion mobility mass spectrometry (IM-MS) was used to identify ions corresponding to the CCL11 tetrameric species bound to Arixtra. Collisional cross sections (CCS) of the CCL11 tetramer-Arixtra noncovalent complex were compared to theoretical CCS values calculated using a preliminary structure of the complex deduced using X-ray crystallography. Experimental CCS values were in agreement with theoretical values, strengthening the IM-MS evidence for the formation of the noncovalent complex. Tandem mass spectrometry data of the complex indicated that the tetramer-GAG complex dissociates into a monomer and a trimer-GAG species, suggesting that two CC-like dimers are bridged by Arixtra. As development of chemokine inhibitors is of utmost importance to treatment of medical inflammatory conditions, these results provide vital insights into chemokine-GAG interactions.

## 1. Introduction

Chemokines are secreted signaling proteins that are involved in biological processes such as inflammation, lymphocyte homing, and development. Chemokines have been implicated in diseases such as asthma, arteriosclerosis, arthritis, multiple sclerosis, and tumor metastasis [1,2,3,4,5]. Due to the role played by chemokines in disease, the development of inhibitors to chemokine function is an area of significant importance. 

Chemokine-mediated signaling relies on a chemokine’s ability to bind to and activate a receptor, as well as on the formation and maintenance of a chemokine concentration gradient which directs leukocyte chemotaxis; disruption of either of these linked functions leads to the disruption of target cell homing [6]. Chemotactic gradients are formed as secreted chemokines diffuse away from the site of release before binding to cell-surface glycosaminoglycans (GAGs) [7]. The importance of this interaction is demonstrated by the observation that while mutant chemokines with decreased GAG binding activity can still signal receptors *in vitro*, these mutants fail to recruit leukocytes *in vivo* [8]. GAG binding has also been linked to chemokine mulitmerization [8,9,10]. Confirming the linkage between GAG binding and multimerization, chemokine mutants with decreased oligomeric character demonstrated decreased recruitment in an *in vivo* cell recruitment assay, despite the fact that these variants did signal *in vitro* [11].

Chemokines are sub-classified on the basis of the spacing of first two cysteines, with CC and CXC chemokines being the most common. Structurally, chemokines have a disordered *N*-terminus followed by these conserved cysteines, a 3_10_ helix, three antiparallel β-strands, and a *C*-terminal α-helix [12]. Though some chemokines appear to exist only as monomers, those that oligomerize tend to do so in a class dependent manner. The dimerization interface of CC chemokines is on the *N*-terminal region, while the first beta sheet is the CXC dimerization interface [12]. CXCL12 has been shown to form both CC-like and CXC-like dimers [13], and chemokine tetramers formed through an *N*-terminal joining of CC-like or CXC-like dimers have been observed [14,15]. In the cases of CCL2 and CXCL10, both a CC-like and CXC-like interface was involved in stabilization of the tetramer [16,17,18,19,20,21]. The GAG binding surface of CCL2 has been mapped, and residues implicated in GAG binding form two patches lining opposing sides of the tetramer. These patches include GAG binding residues from each of two CC dimers, offering a possible explanation for the observation that heparin binding shifts the oligomeric state of CCL2 from dimer to tetramer [8].

In order to explain the details of the linkage of chemokine multimerization and GAG binding, structural studies are needed. Unfortunately, such studies are complicated by the tendency of chemokines to aggregate at high concentration, a tendency only exacerbated by the addition of GAGs. To date, only two chemokine-GAG structures have been solved: those of the chemokine CCL5 in complex with either of two heparin disaccharides [22]. This study, in which crystals were grown at low pH in order to overcome protein aggregation, shows disaccharide binding close to biochemically predicted sites on a CCL5 dimer. Unfortunately, a single disaccharide is far smaller than the expected size of a GAG epitope for chemokine binding [8,10,23,24,25]. Use of longer GAG chains would likely enhance chemokine aggregation and introduce the issue of GAG sequence heterogeneity. While small quantities of homogenous GAG have been purified [26,27], commercially available heparin is available only in size-defined form, and a high degree of sequence heterogeneity exists [10].

In the present study we examine the interaction of CCL11 (Eotaxin-1) and the GAG-like drug Arixtra. Arixtra, a synthetic antithrobin III inhibitor, is a sequence homogenous GAG structurally similar to heparan sulfate, which was available in the quantities sufficient for binding studies. The chemokine CCL11 is a major mediator of inflammation in the lung and has been shown to play a key role in allergic reactions including asthma and parasitic response [28]. NMR of CCL11 indicates that it exists in a monomer-dimer equilibrium under physiological conditions [29]; higher order multimers have not been reported.

The binding of CCL11 to Arixtra was examined first by isothermal titration calorimetry. Following verification that Arixtra does, in fact, bind to CCL11, IM-MS was used to further investigate the noncovalent complex. The CCL11 monomer was analyzed under both denaturing and native conditions to validate preservation of protein native structure in the gas phase. After establishing the feasibility of using mass spectrometry to structurally characterize CCL11, IM-MS was used to analyze the CCL11-Arixtra complex using the orthogonal physical properties of mass-to-charge ratio and collisional cross section. These results show that CCL11 not only forms a previously unreported homotetramer species, but this tetrameric species also binds to Arixtra. These results support previous models of GAG-dependent chemokine oligomerization [8,10,30] and suggest that it may be possible to utilize synthetic GAG molecules to modulate chemokine function *in vivo*.

## 2. Results and Discussion

### 2.1. Binding of CCL11 to Arixtra

In order to confirm the interaction between CCL11 and Arixtra and measure the binding constant (K_D_) arising from this interaction, isothermal titration calorimetry was performed by titrating Arixtra into a CCL11 solution. The dissociation constant of this interaction was shown to be 2.71 ± 0.284 μM (Figure S1), which is similar to the previously determined K_D_ for the CCL7-Arixtra interaction [31]. The monomeric chemokine CCL7 had an n, or number of binding sites, of 0.90 ± 0.03, while CCL11 had an n of 0.78 ± 0.01. This n value is closer to that determined by ITC for the multimeric CCL2, which had an n of 0.70 ± 0.02 and a K_D_ of 7.98 ± 0.77 μM (Figure S1). Previous analysis of the multimeric state of CCL2 by analytical ultracentrifugation, in the presence and absence of added GAG, demonstrated that CCL2 exists in monomer-dimer equilibrium in the absence of GAG and in monomer-dimer-tetramer equilibrium when in the presence of saturating GAG [8]. Thus, the experimentally-determined n value for Arixtra of ~0.7 for CCL2 and CCL11 binding may reflect binding across a variety of chemokine oligomeric states.

### 2.2. Ion Mobility Mass Spectrometry of CCL11 Monomer

**Figure 1:**
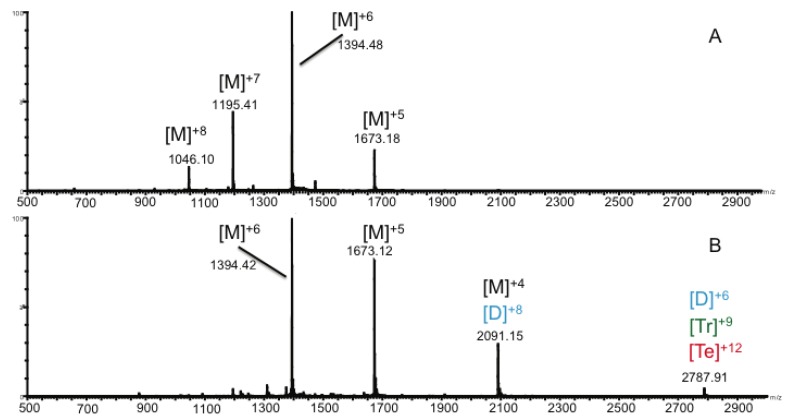
Mass spectra of CCL11 under (**A**) denaturing conditions (50% acetonitrile, 0.1% formic acid) and (**B**) native conditions (250 mM ammonium acetate). M = monomer; D = dimer; Tr = Trimer; Te = Tetramer.

CCL11 was first analyzed with IM-MS in denaturing solvent conditions (Figure 1A), and the resulting spectrum indicated a typical charge state distribution for a protein monomer and confirms the molecular mass. Under native conditions (Figure 1B), a shifted charge envelope with a smaller charge state distribution typical of folded structures, was observed. Collisional cross section (CCS) values were derived from experimental arrival time distributions (ATD) for CCL11 monomer (RCSB Protein Data Bank ID 1EOT) and were compared with theoretical values. Under native conditions, a CCS value of 947 Å^2^ was observed for the [monomer]^5+^ species. MOBCAL [32,33], an algorithm that uses X-ray crystallography and NMR data to predict CCS values, was then used to predict the CCS of the CCL11 monomer using both the projection approximation (PA) and exact hard spheres scattering (EHSS) models. Though the PA model typically underestimates and the EHSS model overestimates CCS [34], values derived from these two models are useful in that they provide boundaries for experimental CCS, *i.e.*, experimental values should be bracketed by theoretical values. Under native conditions, the CCS for the CCL11 [monomer]^5+^ falls within the boundaries established by the PA (918 Å^2^) and EHSS (1129 Å^2^) models for the CCL11 monomer. The experimental CCS value for the CCL11 [monomer]^5+^ is also consistent with the previously published CCS value of 966 Å^2^ observed for the CCL11 [monomer]^5+^ [34]. However, when the CCL11 monomer was analyzed under denaturing conditions, a CCS value of 673 Å^2^ was observed for the 5+ species. This suggests that denaturing solvents induce a collapse in protein structure. If either the solvent composition or instrumental parameters influenced the gas phase structure in such a way that native structure was disrupted, it is expected that the CCS values for monomer would fall outside of the PA/EHSS boundaries. Results for the [monomer]^5+^ lend credence to the preservation of CCL11 native structure in the gas phase under the conditions used in these experiments and further demonstrate the feasibility of analyzing native protein structure using IM-MS [35,36,37,38].

### 2.3. Ion Mobility Mass Spectrometry of CCL11 Tetrameric Species

**Figure 2:**
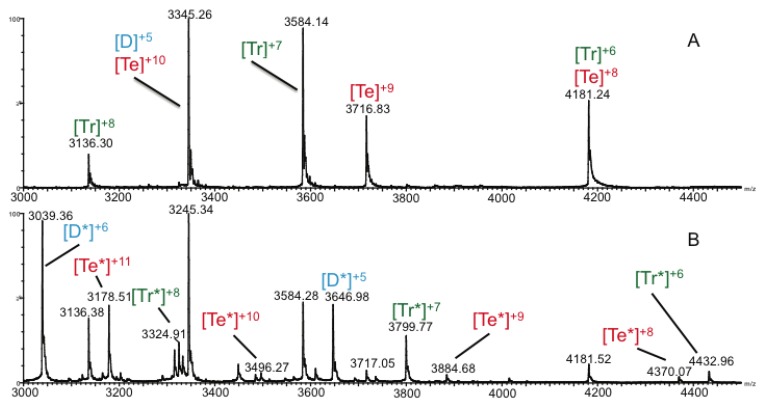
(**A**) Mass spectra of (**A**) CCL11 and (**B**) CCL11+Arixtra magnifying the 3,000–4,500 *m/z* range by a factor of 93 (**A**) and 145 (**B**), respectively. Formation of the CCL11 tetramer-Arixtra complex is supported by the presence of peaks corresponding to multiple charge states of the complex at 3,178, 3,584, 3,885, and 4,370 *m/z*. Higher order oligomers were not observed. D = dimer; Tr = trimer; Te = tetramer; * = species bound to Arixtra.

Evidence for the presence of the oligomeric homo-tetramer was clearly visible in the mass spectrum of the native oligomer shown in Figure 2A, while adduction of the drug Arixtra, and subsequent complex formation was visible in Figure 2B. While monomeric species were the most intense peaks observed in mass spectra of CCL11 (Figure S2), higher order multimers were observed, albeit with lower intensities, at the higher *m/z* range. In addition to the unique 9+ charge state of the tetramer observed in Figure 2A, the 8+ and 10+ charge states were also detected. These latter two charge states overlap with the 6+ trimer and 5+ dimer, respectively. Given the fact that these chemokines are homo-multimers, it is not surprising that the mass-to-charge ratios of the various oligomers overlap; *i.e.*, an 8+ dimer will appear in the same mass-to-charge ratio vicinity of the spectrum as the 4+ monomer. High-resolution mass spectrometry or ion mobility is required to determine the presence or absence of the various overlapping species. In this particular case, IM-MS was used to clearly delineate the presence of each of the oligomers as indicated in Table 1, Figure 1 and Figure 2, and Figure S3. 

**Table 1:** Mass-to-charge ratios, charges, intensities, and assignments of peaks observed in the mass spectrum of the equimolar mixture of CCL11 and Arixtra. Multimeric species were determined by ion mobility mass spectrometry (IM-MS).

Mass-to-Charge (Experimental)	Mass-to-Charge (Theoretical)	Intensity (Counts)	Charge	Species
1,394.4	1,394.4	4.47E6	6	Monomer
1,645.8	1,645.6	8.25E4	6	Monomer + Arixtra
1,673.1	1,673.1	2.53E6	5	Monomer
1,974.8	1,974.5	3.73E4	5	Monomer + Arixtra
2,091.2	2,091.1	1.24E6	4	Monomer
			8	Dimer
2,279.8	2,279.5	1.11E5	8	Dimer + Arixtra
2,389.8	2,389.7	7.51E3	7	Dimer
2,468.2	2,467.9	1.87E4	4	Monomer + Arixtra
2,605.3	2,605.0	1.28E4	7	Dimer + Arixtra
2,787.9	2,787.8	1.79E5	6	Dimer
			9	Trimer
			12	Tetramer
2,913.9	2,913.4	5.83E3	12	Tetramer + Arixtra
2,955.6	2,955.3	1.12E4	9	Trimer + Arixtra
3,039.4 3,041.5 3,136.4	3,039.0 3,041.2 3,136.2	2.97E4 8.50E3 1.18E4	6	Dimer + Arixtra
			11	Tetramer
			8	Trimer
3,178.5	3,178.2	1.42E4	11	Tetramer + Arixtra
3,324.9	3,324.6	7.29E3	8	Trimer + Arixtra
3,345.3	3,345.2	2.09E4	5	Dimer
			10	Tetramer
3,495.9	3,495.9	1.82E3	10	Tetramer + Arixtra
3,584.3	3,584.1	1.47E4	7	Trimer
3,647.0	3,646.6	1.44E4	5	Dimer + Arixtra
3,717.0	3,716.8	2.27E3	9	Tetramer
3,800.0	3,799.4	8.58E3	7	Trimer + Arixtra
3,884.7	3,884.2	1.47E3	9	Tetramer + Arixtra
4,181.5	4,181.3	3.37E3	6	Trimer
			8	Tetramer
4,370.1	4,369.6	1.10E3	8	Tetramer + Arixtra
4,433.0	4,432.4	2.06E3	6	Trimer + Arixtra

Dimerization is not surprising given the fact that monomer, dimer, and tetramer for other chemokines (specifically CCL2) are known to exist in equilibrium *in vivo* [8]. As CCL11 has been previously described to exist in monomer-dimer equilibrium [29], it was expected that the monomeric and dimeric species might be the most abundant in the spectra. However, tetrameric species are also distinctly evident in Figure 2, and the low intensity of the tetramer relative to the monomer is at least partially due to the difficulty associated with desolvating the tetrameric assembly using spray conditions that preserve native structure. Observation of the CCL11 trimer, as observed in Figure 2, is likely due to dissociation of the tetramer under the conditions used since odd numbered oligomers of similar chemokines have not been previously reported [16,39,40]. In a similar vein, there are no reports of biologically-active trimeric chemokines either. As tetramer formation resulting from non-specific oligomerization was a concern, control experiments were performed with the chemokine CXCL8 (interlukin-8) using identical sample and instrument conditions. CXCL8 has been reported to exist in a monomer-dimer equilibrium [41], and the observation of only monomeric and dimeric species (Figure S4) suggests that the MS conditions and concentration of CCL11 were not sufficient to induce non-specific oligomerization.

**Figure 3:**
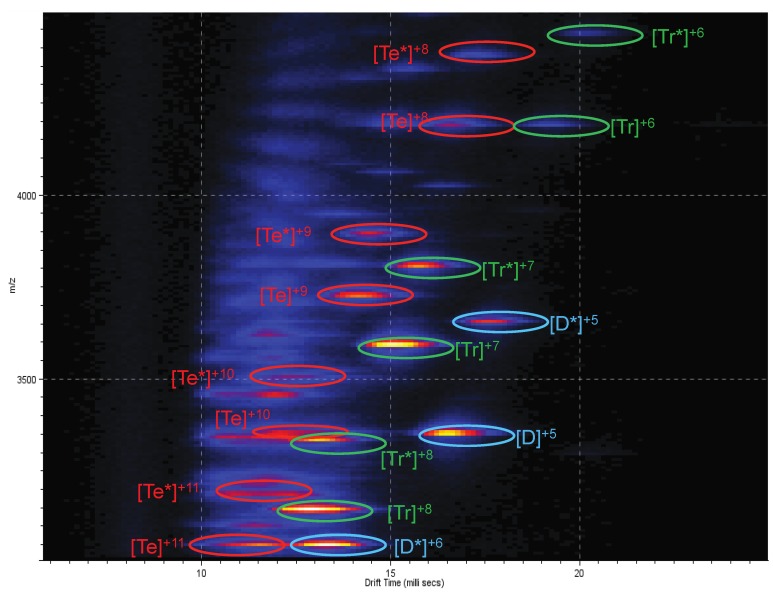
Heat map plotting *m/z* as a function of drift time for the CCL11+Arixtra mixture. Mass range is limited to the 3,000–4,500 *m/z* range illustrated in Figure 2. Ion mobility separation allowed for the characterization of the peak at *m/z* 3,345 as both [D]^5+^ and [Te]^10+^ species. D = dimer; Tr = trimer; Te = tetramer; * = species bound to Arixtra.

Upon analysis of an equimolar mixture of CCL11 and Arixtra, the spectrum shown in Figure 2B was generated. Adducts between the drug and chemokine were evident for the dimer and tetramer. In most instances, adduction of the drug to the chemokine was sufficient to unambiguously separate the homo-multimers, which were overlapping in Figure 2A; *i.e.*, peaks for the dimer/tetramer at *m/z* 3,345 now resolve into [Te*]^10+^ and [D*]^5+^ at *m/z* 3,496 and 3,647, respectively. A heat map plotting *m/z* as a function of drift time for the CCL11-Arixtra mixture is shown in Figure 3. The ion mobility separation of multiple species at a particular mass-to-charge ratio lends confidence to assignments of species with similar *m/z* values such as [D]^5+^/[Te]^10+^, [Te]^11+^/[D*]^6+^, and [Tr]^6+^/[Te]^8+^. These IM-MS data are the first reports showing CCL11 to exist as a tetramer upon drug binding.

**Figure 4:**
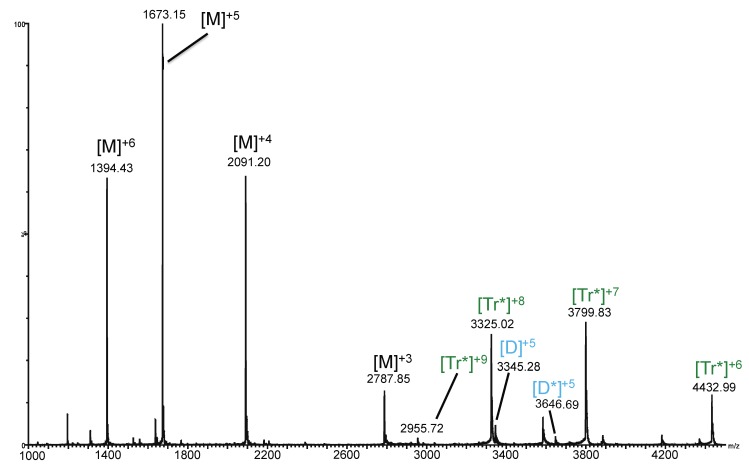
Tandem mass spectrometry of the [Te*]^12+^ peak at 2,913 *m/z* showing that as the Te* complex is fragmented, it dissociates into Tr* and M species. M = monomer; D = dimer; Tr = trimer; * = species bound to Arixtra.

To further investigate the structure, Figure 4 illustrates MS/MS experiments performed on the 12+ charge state of the CCL11 tetramer-Arixtra complex observed at *m/z* 2,913 (Table 1). When subjected to increased collision energy, the complex dissociates into a series of ions corresponding to the monomer and ions corresponding to the trimeric species bound to Arixtra. It is worth noting that charge is conserved in this spectrum; for example, the Te*^12+^ species dissociates into Tr*^6+^ and M^6+^, Tr*^7+^ and M^5+^, *etc*. Primarily trimeric species are observed with the Arixtra bound, suggesting that the mechanism of dissociation of the noncovalent complex is not random. It should be noted that low intensity peaks (<5% of base peak intensity) corresponding to the D^+5^ and D*^+5^ species were observed. 

As a tetrameric assembly of CCL11 has not been previously described, IM-MS data were initially compared to the tetrameric structure of CCL2, a CC-type chemokine, which has been reported to form tetrameric species and exhibited an n value similar to CCL11 using ITC. To justify this comparison, monomeric amino acid sequences of both CCL2 and CCL11 were compared using BlastP [42,43]. Results from this comparison (total score = 107, query coverage = 92%, E value = 7e‒37, max identity = 69%) along with a multiple sequence alignment performed with ClustalW2 [44] (Figure S5A) indicated that the two sequences share a high degree of homology. Similarities between the monomeric structures are also illustrated in Figure S5B, in which the monomeric structure of CCL2 (RCSB Protein Data Bank ID 1DOL [16]) is overlaid with that of CCL11 (RCSB Protein Data Bank ID 1EOT [29]). Structurally, the two proteins appear very similar, with components such as the three antiparallel β-strands and *C*-terminal α-helix from the two chemokines in close proximity to each other. The primary dissimilarities between the two structures occur at the *N*-terminal regions, which is not surprising given that chemokines typically exhibit disordered *N*-terminal regions [12]. 

Given the high degree of homology shared between CCL11 and CCL2, combined with studies in which Arixtra binding to CCL2 homo-multimers has been demonstrated [45], the observation of similar spectral features when analyzing the two chemokines was expected. The CCS value of 947 Å^2^ observed for the CCL11 [monomer]^5+^ species is similar to the 959 Å^2^ value reported for the CCL2 [monomer]^5+^ species [34]. Peaks corresponding to multiple charge states of CCL11 monomer, dimer, trimer, and tetramer were observed in Figure 2; these species were identified as both unbound and bound to Arixtra, which is consistent with a previous study analyzing CCL2-Arixtra interactions [45]. Likewise, one of the more significant features of Figure 3 is that the CCL11 tetrameric species (with Arixtra bound and unbound) tend to aggregate toward the left side of the heat map. Starting with the [Te]^11+^ and ending with the [Te*]^8+^, the tetrameric species are arranged in a nearly linear fashion. Similar linearity in Figure 3 is also observed with the trimeric and dimeric species of CCL11 as well, with the grouping of the trimers to the right of the tetramers and the dimers to the right of the trimers. This pattern of arrangement for the different multimeric species is very similar to that previously reported for the multimers of CCL2 [45], which further suggests that CCL11 forms a homotetramer that binds with Arixtra.

**Figure 5:**
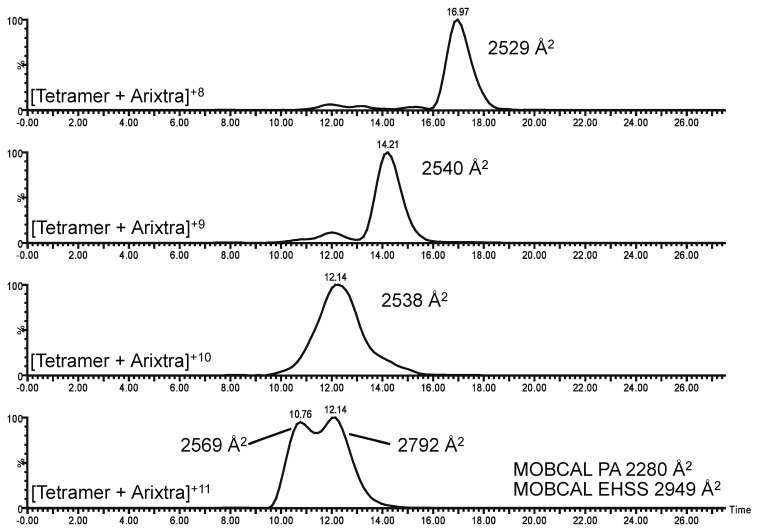
ATDs corresponding to the 8+–11+ charge states of the CCL11 tetramer+Arixtra complex.

After establishing that the CCL11 tetramer-Arixtra complex could be detected in the gas phase, IM-MS data were further analyzed. Figure 5 illustrates experimental ATDs for the 8+–11+ charge states of the CCL11 tetramer-Arixtra complex. CCS values were calculated based on the ATD data. A single primary conformation is observed for the 8+–10+ charge states of the complex, though two conformations are observed for the 11+ charge state, suggesting that the complex is starting to unfold at the higher charge state. It is also noteworthy that the experimental CCS values determined for the 8+–10+ charge states of the CCL11 tetramer-Arixtra complex fall within the PA/EHSS boundaries for the CCL2 tetramer (2,155 Å^2^ and 2,792 Å^2^, respectively). That the CCS values for the 8+–10+ charge states of the CCL11 tetramer-Arixtra complex fall within the PA/EHSS boundaries of the CCL2 tetramer structure provide further supporting evidence that native structure has been preserved in the gas phase (CCS values for the dimer [dimer]^7+^ and [dimer + Arixtra]^7+^ were 1,573 Å^2^ and 1572 Å^2^, respectively, indicating that Arixtra binding has little impact on CCS). This leaves little doubt that CCL11 tetramerizes and binds to Arixtra. Though the boundaries established by the PA and EHSS models are relatively wide for the tetrameric species, experimental values for the 8+–10+ charge states fall near the midpoint of these values. CCS values derived through the PA and EHSS models differ by over 600 Å^2^, calling attention to the need for further developments in informatics pertaining to theoretical CCS determination. The recently described projected superposition approximation (PSA) [46] may offer a more accurate prediction of oligomeric CCS values compared to the PA and EHSS models and will be examined in future studies.

**Figure 6:**
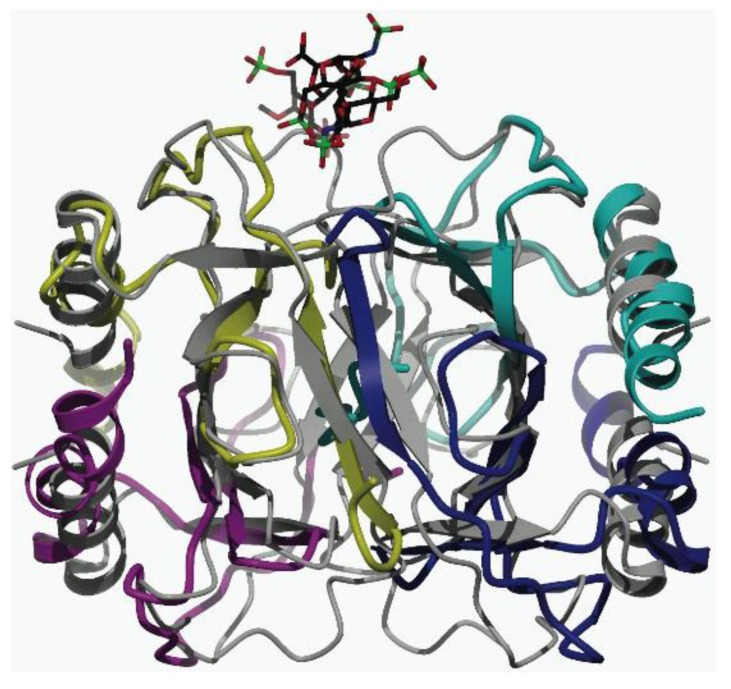
Preliminary X-ray crystallography structure of the CCL11 tetramer-Arixtra complex (shown in color) overlaying the structure of the CCL2 tetramer (1DOL, shown in gray). The Arixtra molecule is shown top center. CCL11 subunits one and two are lower right and upper right, respectively, while subunits three and four are lower left and upper left, respectively. CXC-type dimers are vertically related while CC-type dimers are diagonally related.

An initial attempt was made to determine the X-ray crystal structure of the CCL11 tetramer bound to Arixtra. Figure 6 shows our very preliminary X-ray crystallography structure of the CCL11 tetramer bound to Arixtra and suggests formation of a homotetramer similar to that of the homologous chemokine CCL2 (RCSB Protein Data Bank ID 1DOL) [16]. De-twinning techniques have been used on our preliminary structure, though further refinement of the structure is needed in order to deposit into the RCSB Protein Data Bank. However preliminary, these data clearly support formation of the tetrameric structure, and it is meant to support the CCS data, which also indicate a tetrameric assembly. The current refinement (R_factor_ = 17.6%, R_free_ = 26.7%, additional statistics in Table S1) shows a tetramer with at least one clear Arixtra binding site. Figure 6 shows the two CC-like dimer interfaces between monomers 1 and 4, and between monomers 2 and 3. The CXC-like contacts are between subunits 1 and 2 and subunits 3 and 4. This structure is quite similar to the crystallographic tetramer of un-liganded CCL2 (gray overlay). The theoretical CCS values for this structure (PA = 2,280 Å^2^, EHSS = 2,949 Å^2^) are similar to those calculated using the structure of the CCL2 tetramer, and experimental CCS values determined for the 8+–10+ charge states of the CCL11 tetramer-Arixtra complex fall well within this theoretical range. This further strengthens the argument that the tetrameric form of CCL11 may be favored in solution.

Biologically, the observation that CCL11 forms a tetramer is significant. Various assays have shown CCL11 to exist as monomer or dimer in the absence of GAG [10,29,30]. The presence of Arixtra has been shown to shift this equalibria towards the dimer as seen with analytical ultracentrifugation [47], to increase heterodimerization with CCL2, and to necessitate heterodimerization with CCL8 [30]. These results were initially confusing, as the presumed dimer interface for CC chemokines is near the *N*-terminus and distal to the GAG binding site for CCL11, CCL8, or CCL2 [8,16,48,49]. If, however, upon tetramerization the dimers formed a third interface bridging the GAG, the CC, or CXC interfaces would be important not in GAG binding, but rather for tetramer formation. Such an interface would indirectly stabilize the GAG binding site. The GAG-stabilized dimers would presumably be those observed as GAG-bound CCL11 heterodimers [47]. This shift in a chemokine’s observed dimer interface would not be unprecedented, as heterodimers of the CXC chemokines CXCL8 and CXCL4 form along the CC interface [50]. The location of the GAG-induced heterodimer interface is important because if it is indeed a GAG-bridged interface, heterodimers bearing a composite GAG binding site may potentially have different specificity than the GAG binding sites formed by either GAG-bridged homodimer.

The biological linkage of tetramer formation and GAG binding is supported by the observations that two different classes of mutant chemokines fail to signal *in vivo* [8,11]. Chemokines with a mutation resulting in disruption of their functional CC or CXC multimerization interface recruited cells *in vivo* just as poorly as mutant chemokines with mutations in their basic GAG binding residues, even though both classes of mutants were able to signal *in vitro*. This study provides the first evidence of CCL11 tetramerization, which may be a biologically active form of the chemokine. These *in vivo* tetramers are not readily observable due to the unfortunate propensity, shared by many chemokines, for aggregation or precipitation in the presence of GAG. Though analytically inconvenient, chemokine precipitation on GAG, specifically cell surface heparan sulfate, could provide an effective means for long-term chemokine localization and chemotactic gradient maintenance. 

Heparin and low-molecular weight heparin (LMWH) have been clinically used as anti-clotting and anti-inflammatory applications for decades. Unfortunately, both have been associated with serious side effects, including death, associated with heparin-induced thrombocytopenia. Use of LMWH at low dose minimizes, but does not eliminate, the risk of side effects [51]. The synthetic heparin-like pentasaccharide drug Arixtra has shown anti-clotting activity without heparin-induced thrombocytopenia [52], and it has also been shown to decrease chemokine concentration and disrupt chemokine binding in human plasma [53]. Arixtra contains the rare 3-O sulfation that has been shown to be critical for this anti-clotting activity [54]. Thus, heparin-like drugs, which show neither anti-clotting activity nor risk of heparin-induced thrombocytopenia, are attractive alternatives. If the GAG sequences to which certain chemokines or groups of chemokines bind were known, it should then be possible to selectively inhibit certain chemokine-dependent immune responses and the diseases with which they are associated. The determination of GAG binding sequences is the critical first step in the possible development of such drugs. If the 3-O sulfate is not, in fact, required for Arixtra binding to CCL11, a small, Arixtra-like, synthetic GAG without the 3-O sulfate may be useful as a small molecule therapeutic. In the case of CCL11, which is a major mediator of the leukocyte influx involved in asthma, a synthetic GAG may, for example, be useful as a non-steroidal anti-inflammatory if applied through an inhaler. Further research aimed at studying the interaction of small molecule GAG to various chemokines involved in inflammation are currently underway.

## 3. Experimental

### 3.1. Materials

Arixtra^TM^ (fondaparinux sodium) was purchased from GlaxoSmithKline (Zebulon, NC, USA) and was desalted by dialyzing against water with a 1 kDa molecular mass cutoff Dispo-Biodialyzer (The Nest Group, Southborough, MA, USA). All the other chemicals were purchased from Fisher Scientific and used without further purification.

### 3.2. Expression of Human CCL11 and Sample Preparation

Human CCL11 was expressed and purified as previously described [10]. Briefly, the vector pET 21a including codon optimized CCL11 with the sequence MGSSH_6_ENLYLVPR-wt CCL11 was transformed into TAP302 *E. coli*. Cells were grown at 30 °C and induced with 0.5 mM IPTG at an A600 of 0.4. 1 h after induction, 10 mg/L rifampicin was added. Cells were harvested at 4 h post-induction by centrifugation followed by resuspension in 20 mM Tris-HCl (pH 8), 400 mM NaCl, 20 mM imidazol, and 5 mM MgCl_2_. DNase A was added and cells were frozen at −80 °C. Cell pellets were thawed and lysed by sonication. Lysate was clarified by centrifugation and filtered before application to a Qiagen (Valencia, CA, USA) Ni-NTA column. The column was washed thoroughly with lysis buffer and eluted with 400 mM imidazol in 20 mM Tris-HCl (pH 8), 400 mM NaCl, and 5 mM MgCl_2_. Eluate was dialyzed against 20 mM Tris (pH 8), 300 mM NaCl, 3 mM CaCl_2_ and then digested with thrombin at 1:1,000 protease:total protein at room temperature for 72 h. Digested CCL11 was further purified by C_18_ reverse phase HPLC over a 25%–75% acetonitrile gradient. HPLC fractions were lyophilized and stored at −80 °C. For isothermal titration calorimetry experiments, lyophilized CCL11 was dissolved into 50 mM potassium phosphate (pH 7.0) then dialyzed against 10 mM Tris-HCl (pH 8.0), 10 mM NaCl. For IM-MS experiments, lyophilized CCL11 was dissolved in 250 mM ammonium acetate (pH 6.8) and desalted using three successive spins with a Bio-Rad P-6 Bio-Spin chromatography column (Hercules, CA, USA).

### 3.3. Isothermal Titration Calorimetry

Arixtra (1 mM) was titrated into a 50 μM solution of CCL11 with both solutions in 10 mM potassium phosphate (pH 7.5), 100 mM NaCl at 25 °C. Titration of Arixtra into CCL11 was one injection of 1 μL followed by 44 injections of 5.999 μL, with a 60 s initial delay and 250 s between injections. Heats of injection were recorded using the MCS-ITC (MicroCal, Northampton, MA, USA) and processed using Origin version 2.9 (OriginLab, Northampton, MA, USA), which reported the *K_d_*, ΔH, and *n* (number of binding sites) of the chemokine-Arixtra interaction.

### 3.4. Ion Mobility Mass Spectrometry

IM-MS data were acquired on a Waters Synapt G2 HDMS (Milford, MA, USA). This instrument utilizes a traveling wave ion mobility cell previously described [55]. Samples were introduced into the instrument with nanospray emitters prepared from 1 mm O.D., 0.78 mm I.D. borosilicate glass capillaries (Sutter Instrument Company, Novato, CA, USA) using a Sutter P-97 Flaming/Brown micropipette puller. Emitters were gold-coated using a Quorum Technologies SC740 sputter coater (Ringmer, East Sussex, UK). CCL11 was sprayed at a concentration of 20 μM, and CCL11:Arixtra mixtures were sprayed at a 1:1 molar ratio.

The instrument was operated with the following parameters: capillary voltage, 0.78 kV; sample cone, 17 V; trap collision energy, 40 V; transfer collision energy, 0 V; trap DC bias, 45 V; trap gas flow, 4 mL/min (Ar); IM-MS gas flow, 90 mL/min (N_2_). These conditions were kept as low as possible in order to preserve protein native structure. The backing pressure was set to 6.5 mBar by regulating the backing Scroll pump with a Speedi valve. IM-MS wave velocity was set to 725 m/s, and the IM-MS wave height set to 40 V. A 32 kV radio frequency generator was used to supply voltage to the quadrupole. LM resolution was set to 4.7, and HM resolution was set to 15. Data were acquired for 5 min with a mass range of 500–8,000 *m/z*, and the time of flight analyzer was calibrated with a solution of cesium iodide (50 μg/μL) over this mass range. Tandem mass spectrometry (MS/MS) data were acquired using a trap collision energy of 3 V and a transfer collision energy of 50 V; otherwise, all other instrument parameters were identical to those used for MS data. IM-MS data were calibrated as previously described [56] using the drift times of the 11+ through 21+ charge states of 10 μM horse heart myoglobin in 50% acetonitrile, 0.1% formic acid. Data were analyzed with MassLynx 4.1. 

## 4. Conclusions

In this manuscript, the existence of the tetrameric assembly of the chemokine CCL11 bound to the GAG Arixtra has been demonstrated. Native MS experiments have shown a continuous charge state distribution of the tetrameric species bound to Arixtra, and IM-MS has allowed for the measurement of ATD values and calculation of CCS values, which both support the existence of a tetrameric assembly. Similarly, very preliminary crystallographic representations of the assembly also illustrate the formation of a CCL11 tetramer noncovalently linked to Arixtra.

## Supplementary Files

Supplementary File 1 Supplementary Materials (DOCX, 728 KB)
