# Supplementary material for: Structural Evidence for the Tetrameric Assembly of Chemokine CCL11 and the Glycosaminoglycan Arixtra™
Source: Biomolecules. 2013 Nov 6;3(4):905–22. doi: 10.3390/biom3040905 (PMC4030970; doi:10.3390/biom3040905)
Supplement: Supplementary File 1 — Supplementary Materials (DOCX, 728 KB) [file biomolecules-03-00905-s001.docx]

**Supplementary Materials for**

Structural Evidence for the Tetrameric Assembly of Chemokine CCL11 and the Glycosaminoglycan Arixtra™

**Figure S1.** Isothermal titration calorimetry of CCR2 ligands in the presence of Arixtra. Upper left CCL2, upper right CCL2 P8A engineered monomer variant, lower left CCL7, lower right CCL11.


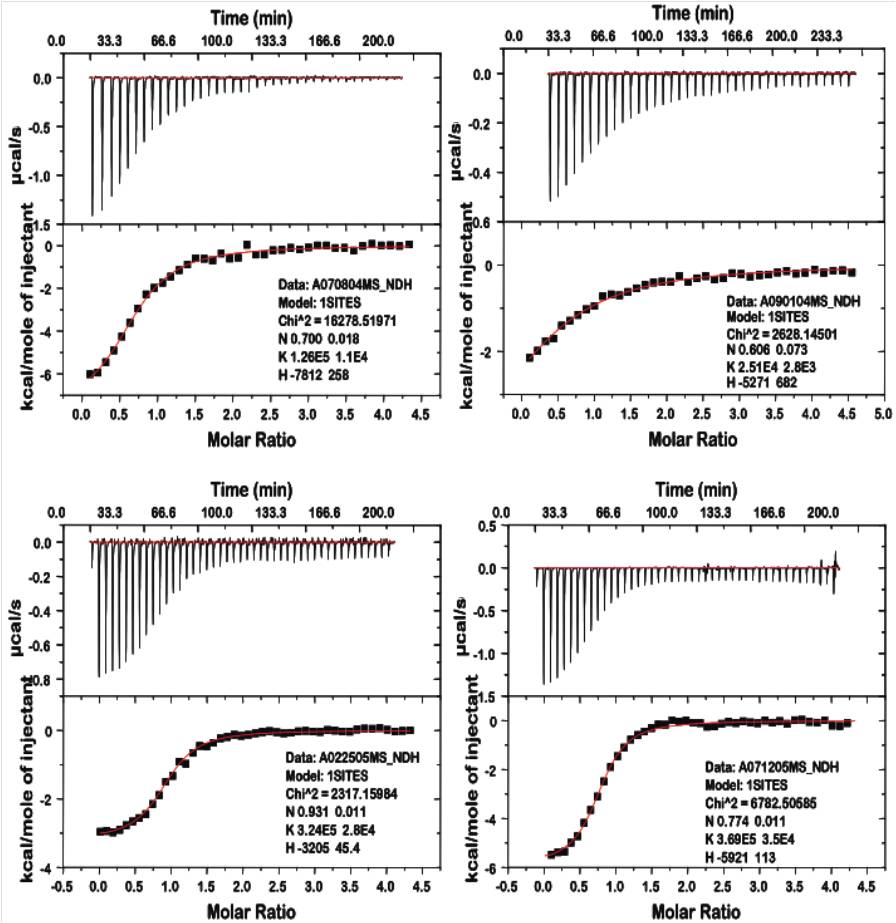


**Figure S2.** Full mass spectrum of CCL11 in the presence of Arixtra, indicating that monomeric CCL11 is by far the most abundant species in the mixture. Intensities of all components of the spectrum are collected in Table 1.


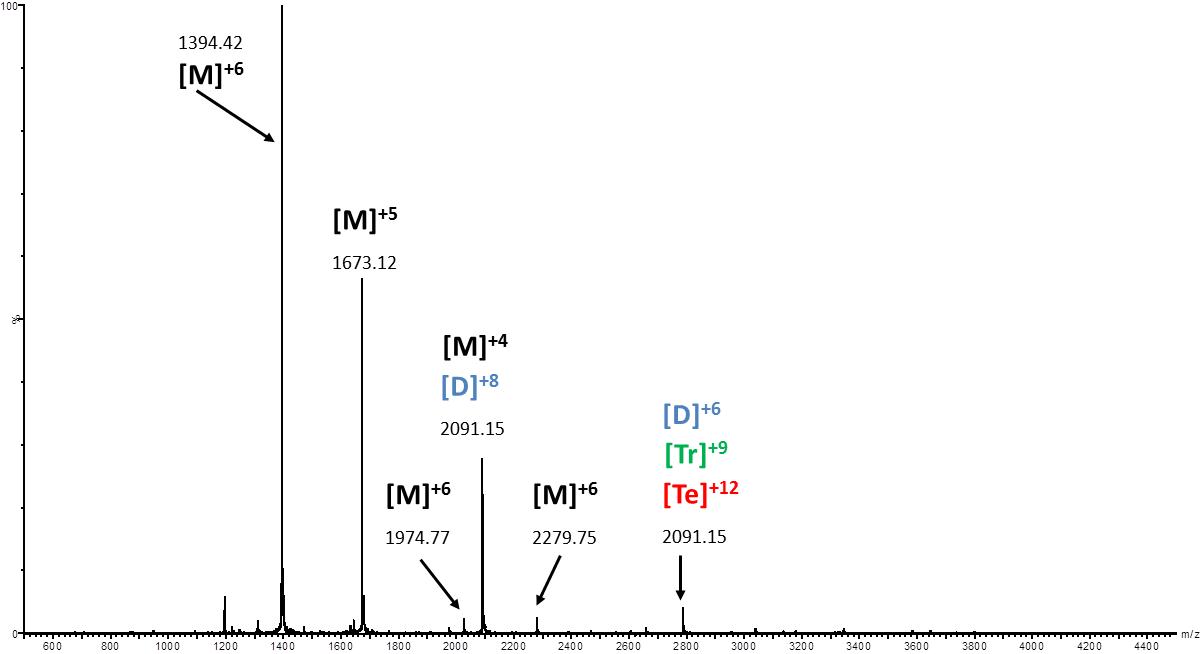


**Figure S3.** Arrival time distributions of CCL11 species with overlapping *m/z* ratios below *m/z* 3,000. These species include (**A**) *m/z* 1,394; (**B**) *m/z* 2,091; (**C**) *m/z* 2,787. Higher *m/z* species are illustrated in Figure 3. While only a single species is observed at *m/z* 1,394 (**A**), multiple species are observed at *m/z* 2,091 and 2,787. Assignment of these components is also strengthened by observation of the all species illustrated below bound to Arixtra (*i.e.*, [M*]^+4^ and [D*]^+8^, Table 1), which have unique *m/z* values.


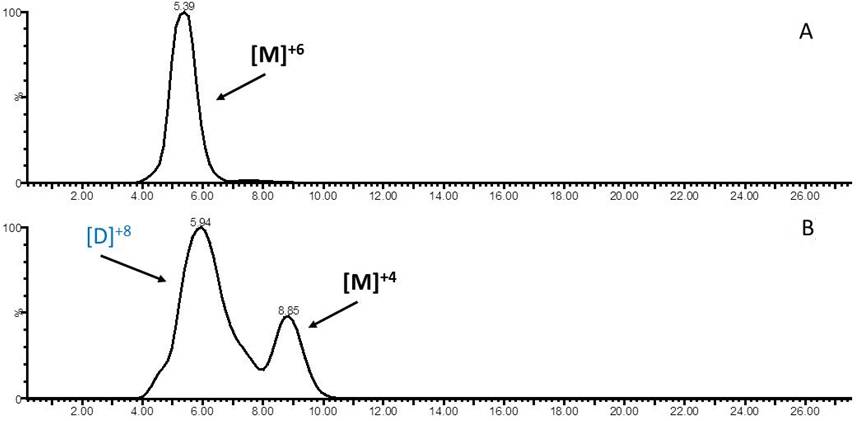


**Figure S3.** *Cont.*


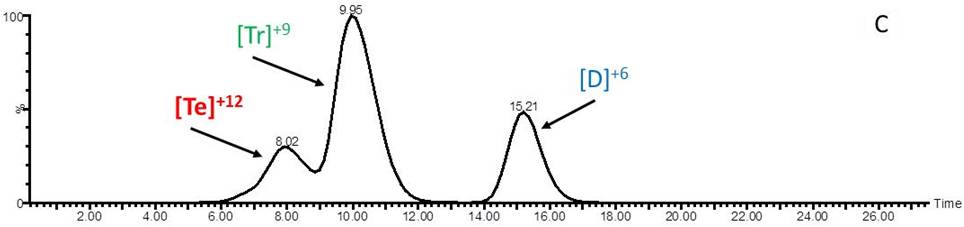


**Figure S4.** (**A**) Mass spectra of CXCL8 showing the 500–3,000 *m/z* range and (**B**) the
3,000–4,500 *m/z* range. (**B**) is magnified by an order of 783.


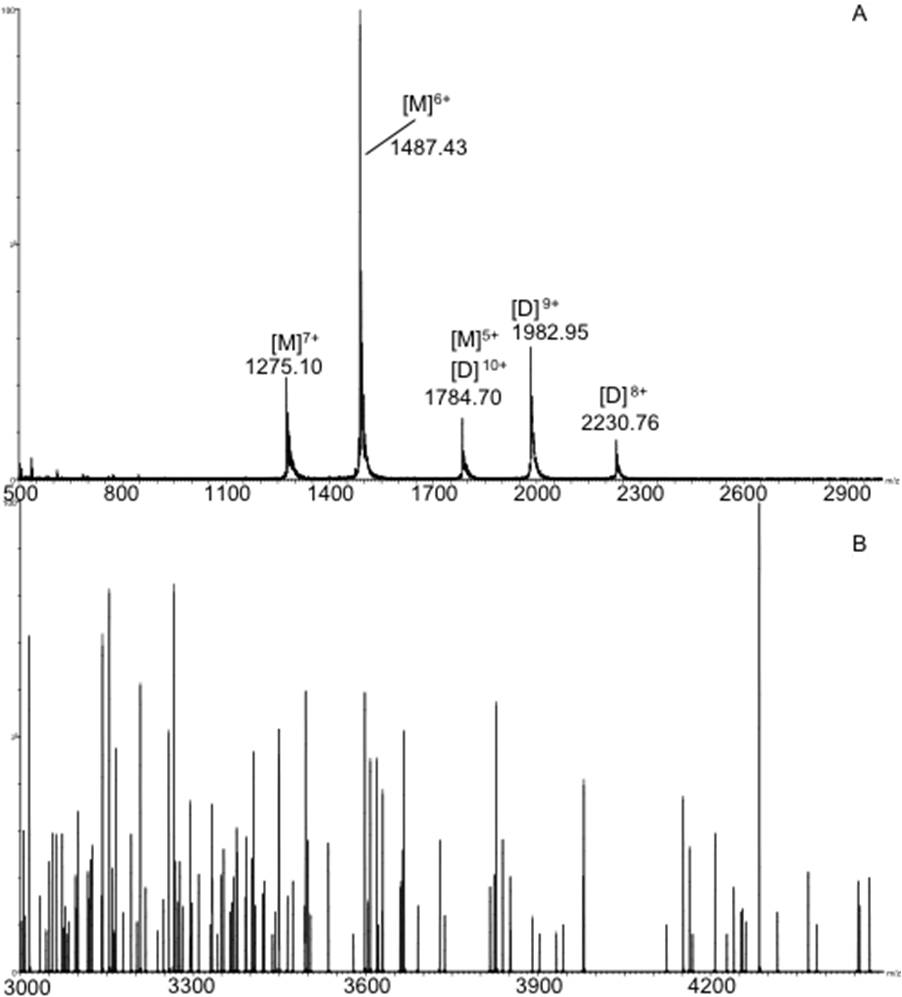


**Figure S5.** (**A**) Multiple sequence alignment of CCL2 and CCL11 and (**B**) overlay of CCL2 (blue) and CCL11 (orange) monomeric structures illustrating homology shared by the two chemokines. In (**A**), ***** indicates fully conserved residues; : indicates strongly similar properties; . indicates weakly similar properties; red indicates small, hydrophobic residues; blue indicates acidic residues; magenta indicates basic residues; and green indicates hydroxyl/sulfhydryl/amine/G residues.


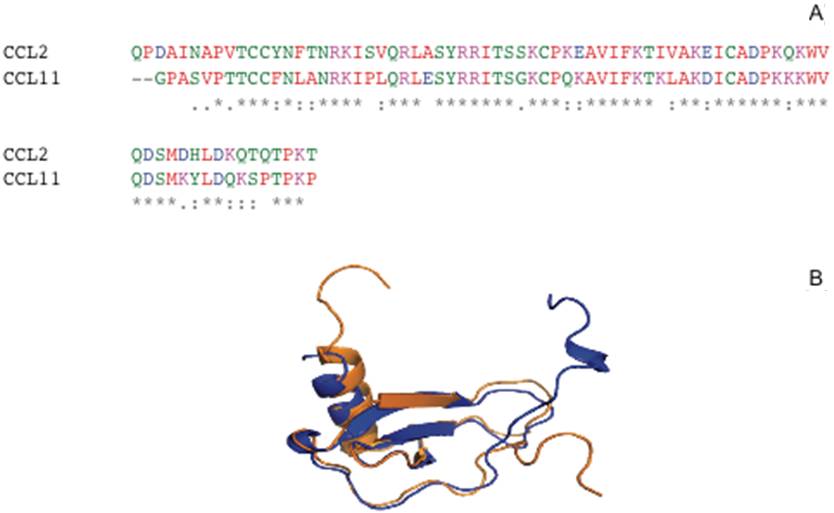


**Table S1.** Data collection, molecular modeling, and refinement statistics for preliminary crystal structure illustrated in Figure 6.

| **Space Group** | **P21** |
| --- | --- |
| Cell | 49.35 69.92 49.47 90 96.81 90 |
| Resolution Limits | 28–2.4 (2.53–2.4) |
| % Data | 96.1 (96.1) |
| I/sigI | 10.7 (2.2) |
| Redundancy | 2.1 (2.0) |
| R_merge_ (%) | 4.5 (32.7) |
| Wilson B | 61 Å^2^ |
| Vm | 2.7 Å^3^/Da (tetramer) |
| 11,783 Reflections, 10,116 Parameters | |
| 517 Reflection in free set | |
| Rfactor | 17.6% |
| Rfree | 26.7% |
| wR2 | 37.4 |
| Goof | 7.5 (restrained 3.1) |
| BASF | 0.486 |
| ASU | 4 Eotaxin monomers (3 complete, 1 lacking 5 *N*-terminal residues), 1 complete Arixtra, 1 PEG-400, 50 Solvent |
